# Path-ATT-CNN: A Novel Deep Neural Network Method for Key Pathway Identification of Lung Cancer

**DOI:** 10.3389/fgene.2022.896884

**Published:** 2022-06-16

**Authors:** Lin Yuan, Jinling Lai, Jing Zhao, Tao Sun, Chunyu Hu, Lan Ye, Guanying Yu, Zhenyu Yang

**Affiliations:** ^1^ School of Computer Science and Technology, Qilu University of Technology (Shandong Academy of Sciences), Jinan, China; ^2^ Cancer Center, The Second Hospital of Shandong University, Jinan, China; ^3^ Department of Gastrointestinal Surgery, Central Hospital Affiliated to Shandong First Medical University, Jinan, China

**Keywords:** ATT-CNN, Path-ATT-CNN, primary lung tumor symptom, pathways, neural network

## Abstract

Attention convolutional neural networks (ATT-CNNs) have got a huge gain in picture operating and nature language processing. Shortage of interpretability cannot remain the adoption of deep neural networks. It is very conspicuous that is shown in the prediction model of disease aftermath. Biological data are commonly revealed in a nominal grid data structured pattern. ATT-CNN cannot be applied directly. In order to figure out these issues, a novel method which is called the Path-ATT-CNN is proposed by us, making an explicable ATT-CNN model based on united omics data by making use of a recently characterized pathway image. Path-ATT-CNN shows brilliant predictive demonstration difference in primary lung tumor symptom (PLTS) and non-primary lung tumor symptom (non-PLTS) when applied to lung adenocarcinomas (LADCs). The imaginational tool adoption which is linked with statistical analysis enables the status of essential pathways which finally exist in LADCs. In conclusion, Path-ATT-CNN shows that it can be effectively put into use elucidating omics data in an interpretable mode. When people start to figure out key biological correlates of disease, this mode makes promising power in predicting illness.

## Introduction

Processing complicated structures and dependencies in data is a difficult job (Ampie et al., 2015). Deep neural network methods offer ways to process the nonlinear model (Bengio et al., 2017). Our goal was to learn many insightful levels of abstraction (Cai and Wei, 2020). Particularly, the attention convolution neural network (ATT-CNN) has obtained achievement (Chen et al., 2020). ATT-CNN has been put into used to solve biological problems because of its nominal grid-structured version in biological collection, and it is difficult for people to solve it (Du et al., 2019). Lately, many articles have used traditional neural networks to interpret omics data collection (Evans et al., 2018). Also, data are offered by the National Cancer Center Research Institute (NCCRI) program which is in the analysis of many molecular parts (Goldstein et al., 2017). Shortage of interpretability is a key part which makes the medical neural networks adoption limited (Golestaneh and Karam, 2017). The trained models or results by biological interpretation are used to understand the complicated human diseases’ biological mechanisms better (Learning, 2020). A method named class activation mapping (CAM) is developed which uses global average pooling. Its task is to increase the interpretability (LeCun et al., 2015). CAM, which makes the image regions visual, is applied by the CNN which precisely generates a target map (Li et al., 2021). There are some limitations to CNN: (i) the CNN architecture that is shown in modeling should be modified, which can be altered by fully connected layers. In addition, the average pooling layer can also alter it. Its goal is to employ CAM, and (ii) CAM of the modified network should be fine-tuned. A more common method was lately developed named as X-gradient-weighted class activation mapping (X-Grad-CAM) (Liu et al., 2019). It uses any target class gradient information which immerses into the convolutional layer. Its task is to generate class activation maps. We created another novel method (Lomonaco et al., 2022) named as Path-ATT-CNN (Nandhini Abirami et al., 2021). This can generate an interpretable ATT-CNN model which uses omics data in cancer outcomes (Wang et al., 2019). Data are input into the ATT-CNN model, and biological pathway images are applied (Wirsching and Weller, 2017). This is made in use of integrated omics data with the low dimensional space including molecular characteristics of lung adenocarcinomas (LADCs) (Wu, 2017). Our goal was to find the biological pathways which are linked with outcomes after modeling. We showed that the method can be used for predicting primary lung tumor symptom in diagnosed patients (Wu et al., 2020) and for differentiating between primary lung tumor symptom (PLTS) and non-primary lung tumor symptom (non-PLTS). The key biomarker identification that is linked with survival in LADC patients can explain the implicit biological performance (Yuan et al., 2018). It plays a vital role in LADCs. In this article, we showed that the ATT-CNN model is better than other methods (Yuan and Huang, 2019), and then, the model applying X-Grad-CAM is interpretable (Yuan et al., 2021a). It makes influential biological pathways visual in identification (Yuan et al., 2017).

## Materials and Methods

### Data

G,C separately show data for LADCs, RNA expression, and M ∈ R_n×r_ originate in the Gene Expression Omnibus. Here, n and r refer to the samples and genes numbers, respectively. Primary lung tumor symptom (PLTS) is defined as presence of lung adenocarcinomas in patients, and non-primary lung tumor symptom (non-PLTS) is defined as absence of lung adenocarcinomas in patients (Yuan et al., 2021b).

### Pathway Image

In order to figure out this problem, data are input into pathway-level profiles, and pathway information is drawn first. The pathways that are enriched by genes of lung adenocarcinomas (LADCs) are identified. A total of 2,200 pathways are shown. Pathway p_i_, the RNA expression which is connected with gene data is drawn from the LADC expression matrix (Yun et al., 2019). The pathway produces a midterm matrix $B\in R^{n\times r_{i}}$ (Yuan et al., 2016), where $r_{i}$ means the quantity of genes. Also, matrix B makes up samples. The sample of the given pathway is given in rows and genes are given in columns. Matrix B resolves uncorrelated components, which is expressed as $G_{\mathrm{pi}}\in R^{n\times q}$ . This task could be finished by using principal component analysis. Here, q means the number of principal components (PCs) (Yuan et al., 2015). The process is duplicated for all 2,200 pathways. It leads to the merged matrices $G_{p}\in R^{n\times 2200q}$ for 2,200 pathways. Last but not the least, for each sample $s_{j}$ , $G_{\mathrm{sj}}\in R^{2200\times q}$ is derived by rearranging the matrix. This process is named according to the sample $s_{j}$ in the image. The rows represent 2,200 pixels. The columns represent q PCs. They are combined for the omics types (Yuan et al., 2017). Finally, they will be put into the ATT-CNN model.

### Sequence of Pathways

X-Grad-CAM is used to sequence the pathways. Its goal was to figure out the key PLTO pathways in LADCs. It can find target regions. So, when reciprocal pathways are linked with pathway images, the possibilities of X-Grad-CAM identifying key pathways are wide. It is also in line with the nature of ATT-CNNs. ATT-CNN is able to localize primary pathways in input position. It is originally rational to make connected pathways. Pathways are in nearness on the pathway images. In this way, people can better find important regions. Therefore, 2,200 pathways are ranked in the conduct: person connection between pathways which is figured out on a matrix of 2,200q is generated. Combining all resultant pathway images makes up the matrix. This process occurs in 2,200 pathways. Pathways lie in among unselected situation. It was the most similar to the pathway in the row. Finally, all pathway images have the identical sequence of pathways.

### ATT-CNN Architecture

**FIGURE 1:**
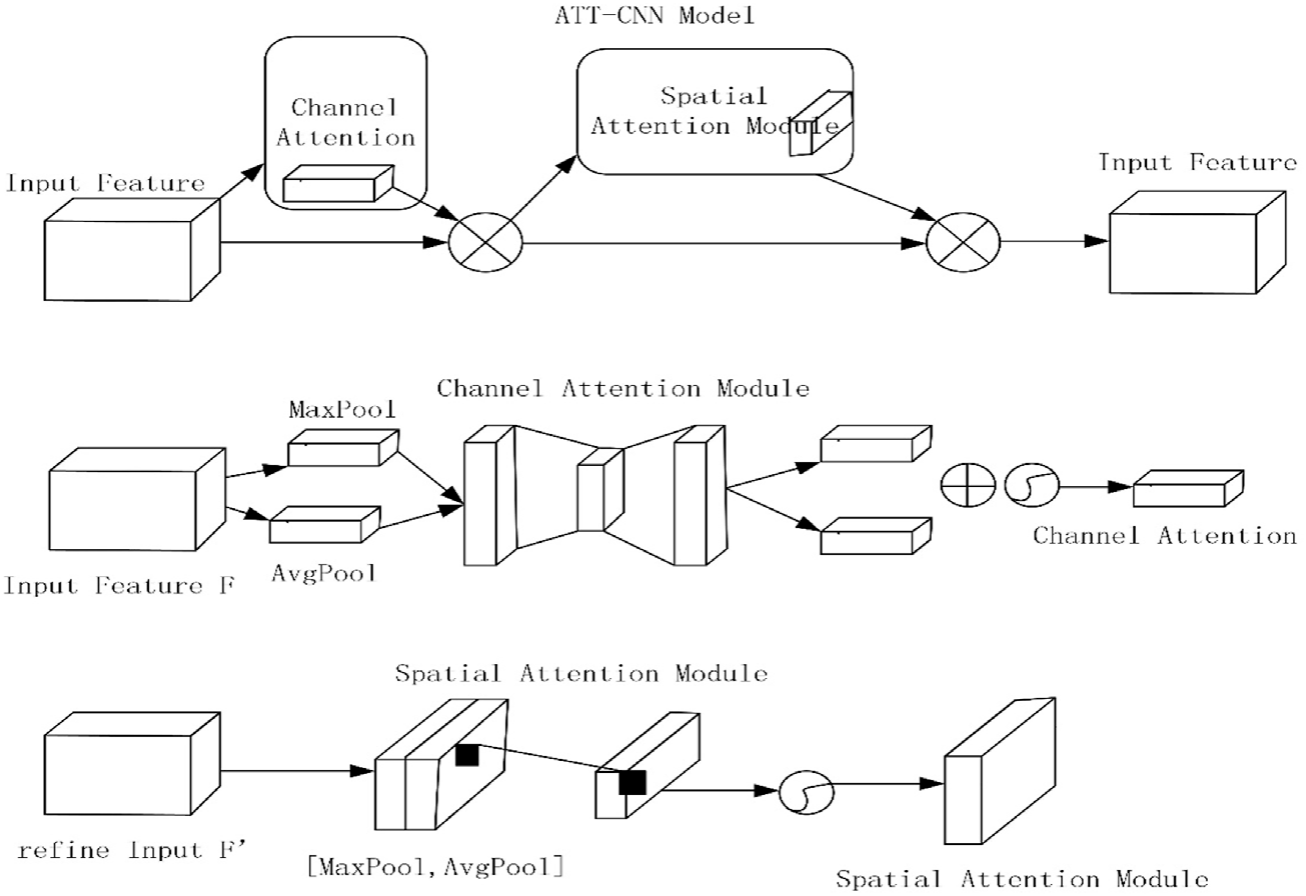
Model of ATT-CNN.

We collect spatial content of a feature map. We make it by applying average pooling and max pooling. This process generates two totally different spatial context terms: $F_{\max}^{c}$ and $F_{\max}^{c}$ . Also, they are put on a shared network. Their goal was to create a channel attention map M_c_∈R^C×1×1^. Multi-layer perceptron (MLP) has one hidden layer which is made up of a shared network. Second, the hidden layer size is set to R^C/r×1×1^. Here, r means the speed of reducing, which can reduce parameter overhead. Finally, we apply element-wise summary. In this way, we can combine the output. Pathway images consists of the input layer. They also have a 2 × 2 size which is followed by average-pooling and max-pooling separately, and then it is combined by the sigmoid function. The dropout rate is 0.5, and activation function is RELU. Also, it is combined when parameters are put into dilapidation convolution, 32 filters, and size of 2 × 2. The output comes from the dropout layer. The output is finally deformed to a vector. It is linked with a fully connected layer which has 64 nodes, which are ensued by a dropout layer and a Softmax layer. SGD is used as an optimizer with a learning rate of 0.001. In this way, we get the final output which is mentioned in this article. Figure 1 illustrates the model of ATT-CNN.

### Cross Validation

The model which uses a five-fold cross validation will be trained under 40 repeats. Data which are randomly assigned as follows undergo five-fold cross validation: 20% of data are designed as a test set. Also, the unexhausted data are allotted as a training set, and the validation set is applied to adjust hyper-parameters. The model performs by using the training collection and optimizing hyper-parameters which are in the ATT-CNN model. It is performed in each cross validation which is assessed on the test collection. Also, the area under the curve is used to test the performance.

### Biological Interpretation Using X-Grad-CAM

Thinking of an input image, I, l means the scale which is in the target visual layer. F^l^ is a score set. Also, it shows the manner of the target layer. $S_{c}\left(F^{l}\right)$ means the class score of score c. This premise that the lth layer holds K is featured with diagrams. In addition, the reaction of the kth feature diagram is shown as $F^{lk}$ . The reaction at position (x,y) in $F^{lk}$ is shown as $F^{lk}\left(x,y\right)$ . X-Grad-CAM, which is for class-discriminative localization mapping, is used to identify key pixel images which are linked with PLTO in LADC patients. We can produce class activation diagrams. To finish this job, we should figure out the gradient of scores ( $F^{lk}$ and S_c_). Each feature diagram consists class c. Neurons which are computed weights ${\text{w}}^{\text{c}}$ for class c can be expressed as:

$$\alpha_{c}^{k}=\sum_{x,y}\left(\frac{F^{lk}\left(x,y\right)}{\sum_{x,y}F^{lk}\left(x,y\right)}\frac{\partial S_{c}\left(F^{l}\right)}{\partial F^{lk}\left(x,y\right)}\right).$$ (1)

In Eq. 2, M_c_ means a sum of weighted feature diagram. This motivation is strength pixels. Its intensities are good for increasing the score. A localization diagram for every class is regulated to exist in between 0 and 1. The class activation diagrams have the identical size, which is on the feature diagram. It is put into the figure of the input:

$$M_{C}^{X-Grad-CAM}\left(x,y\right)=\sum_{k=1}^{K}\left(\alpha_{c}^{k}F^{lk}\left(x,y\right)\right).$$ (2)

**FIGURE 2:**
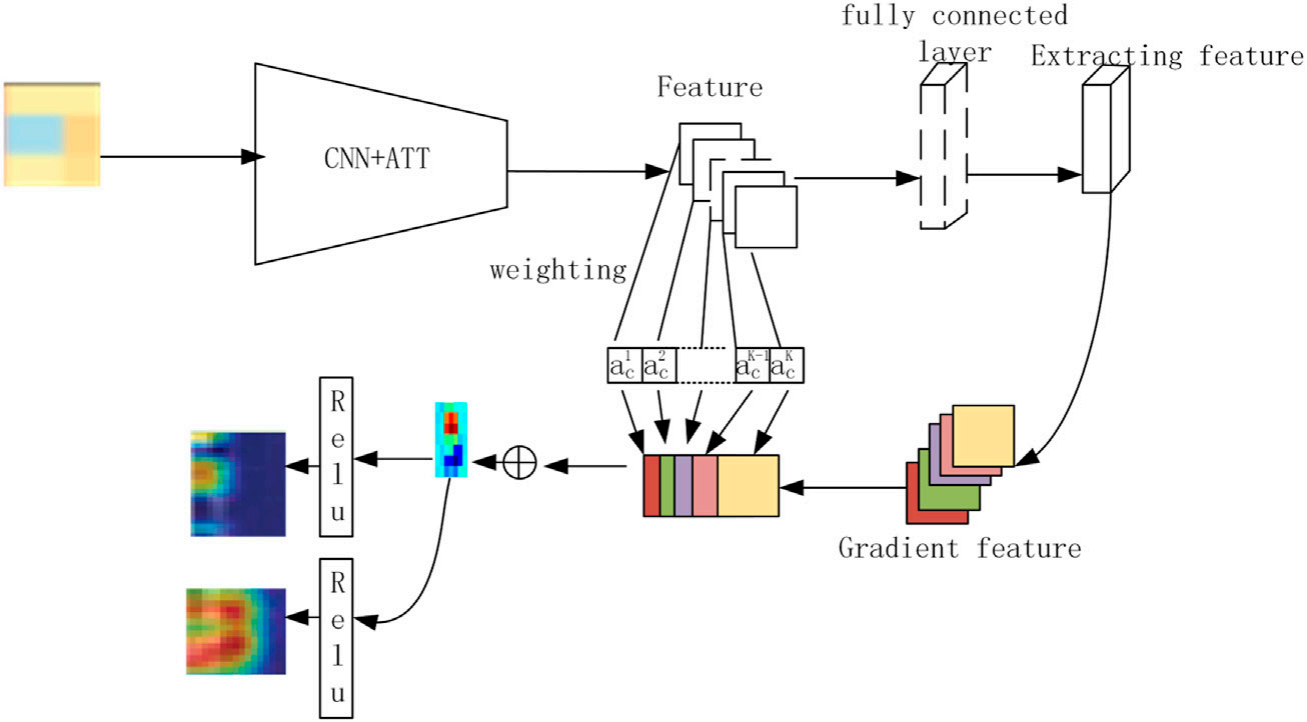
Process of generating the pathway images.

Because of biological interpretation which applies X-Grad-CAM, the ATT-CNN model, which is unsimilar with the model that has cross validation, is made by applying all samples. Every sample, which is input into the model, produces two activation diagrams (PLTO and non-PLTO). Figure 2 explains the process of generating the pathway images.

So, the difference map is produced. A statistical analysis, which duplicates this process for all samples, is applied. Assessing the statistical difference between the PLTO and non-PLTO groups is not an easy job. We use the Wilcoxon rank-sum test to figure out this problem for a given pixel.

### Comparison With Benchmark Methods

Several machine learning methods are compared with the predictive performance of Path-ATT-CNN. The same settings, which are used as described, are shown for all the experiments. Also, the setting is also in the aforementioned cross validation operation. There is a neural network architecture which is empirically adjusted. It composes an import layer. It comprises five hidden layers. It makes up an output layer. There are nodes in each hidden layer whose numbers are 10 k, 7.5 k, 5 k, 2.5 k, and 500. ReLU which acts as an activation function is located in the hidden layers. The number of dropout rate is calculated using the hidden layers and is set at 80%. The manner of MiNet which initially plans to figure out the identical index that is altered is identified by this method. These several famous methods which are trained by the Adam optimizer act as comparison with Path-ATT-CNN.

## Result

### Data

Our goal was to explain molecular characteristics of lung adenocarcinomas (LADCs). A total of 226 primary lung LADCs of medical stages I–II are evaluated. They undergo genome-wide expression identification. In total, 174 genes are chosen as upregulated specifically in 79 lung LADCs. A total of 79 cases are split into: 11 ALK-positive patients, 36 A triple-negative LADC patients, and 32 patients of B triple-negative LADC patients. They are unattended clustering according to the demonstration of 174 genes. Group A triple-negative LADC patients show remarkably worse prognosis for relapse than LADC patients and group B triple-negative LADC cases. A total of 9 genes are authenticated to remarkably put in group A triple-negative LADC cases which are good for finishing their prognoses. Genes, which distinguish this group of LADCs, are suggested to be helpful for patients chosen for additive chemotherapy after operation resection of stages I–II triple-negative LADCs and provide useful insights into the process of molecular target therapies for these patients. The data of LACDs are composed of RNA expression which is assessed at the gene level: RNA expression for 843 genes in 578 cases. There are patients who survived in the last follow-up ≤ 10 months. Also, those patients are removed for finishing the task and receiving better outcomes. Also, PLTO and non-PLTO groups have 155 and 432 cases, respectively. Class weights which are set in the model are important. This method can figure out imbalance data in the light of the ratio of the quantity of samples in the two groups. In total, out of 489 unusual genes we selected 346 TP53. PCA tests have shown individual pathways, respectively, on data type which transfers gene-level information to pathway-level information. The average smoking age in PLTO and non-PLTO groups is 20 and 30 years, respectively. The difference in smoking age in the two groups is remarkable with a *p*-value < 0.001. This method is conducted by applying a two-sample *t*-test. Smoking age plays a key role in the survival. The variable is in the ATT-CNN model. Also, it will be put into the fully connected layer.

### Modeling Performance

**FIGURE 3:**
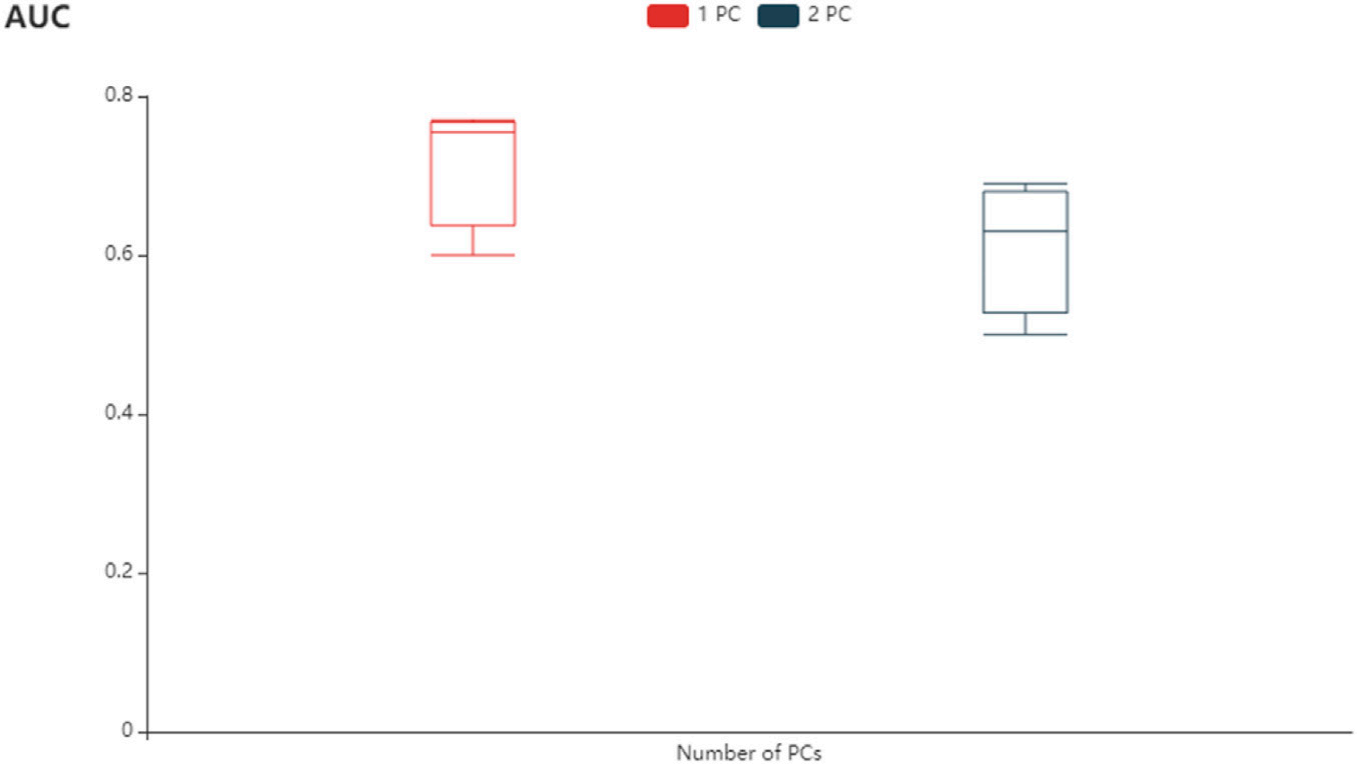
Modeling performance.

The ATT-CNN model which is used to identify PLTO and non-PLTO groups in GSE, which uses pathway images is evaluated in a five-fold CV scheme. In total, 2,200 rows and 3 × q columns are generated by a pathway image representation. Here, q refers to the number of PCs. Also, each row represents the same pathway. In the experiment, the size of the experiment is valued with q = 1. In every experiment, which has an average AUC over 30 iterations of the five-fold CV, is reported by us. The performance of PC (q = 1) is shown in Figure3, which reaches an average AUC of 0.763. The performance of q = 2, a model without smoking age, and the average AUC is 0.697. This situation shows the importance of smoking age. Otherwise, the ATT-CNN model on pathway images with q = 2, which uses a group of two omics types, is conducted. In conclusion, the application of omics types in modeling shows better performance.

### Comparison With Benchmark Methods

There are four different machine learning methods mentioned by us in this article. Also, four methods are compared with the performance of Path-ATT-CNN. The ATT-CNN model is used to identify PLTO and non-PLTO groups in GSE. Class weights are put into the model because the ratio of the quantity of samples is in the two groups. With regard to most cancers, Path-ATT-CNN performed better than any other methods (Yuan et al., 2021a). The details are shown in Table1.

**TABLE 1:** Comparison of predictive performance.

Cancer	Path-ATT-CNN	Logistic regression	SVM with RBF	Neural network	MiNet
LGG	0.857 ± 0.007	0.816 ± 0.036	0.884 ± 0.017	0.791 ± 0.031	0.854 ± 0.027
LADCs	0.657 ± 0.014	0.581 ± 0.028	0.624 ± 0.034	0.573 ± 0.012	0.597 ± 0.042
KIRC	0.719 ± 0.009	0.654 ± 0.034	0.684 ± 0.027	0.563 ± 0.031	0.659 ± 0.030

### Identification of Key Pathways

The ATT-CNN model, which applies samples lying in pathway images, is produced. To finish this, we should use the first two PCs for patient omics types, which is able to make helpful insights into biological interpretations. Mechanisms are linked with survival in LADC patients and self-reliant from smoking age. This is used to classify the biological mechanisms (Yuan et al., 2021a). Individual pathway images are integrated into the experiment. X-Grad-CAM produced two activation maps (LADCs and non-LADCs). $L_{i}^{plto}$ and $L_{i}^{non-plto}$ are the two activations diagram for PLTO and non-PLTO for a given sample $s_{i}$ . When the model is trained better, the possibility of an input sample $s_{i}$ is high. The possibility of an input lies in the $L_{i}^{plto}$ group, and $L_{i}^{plto}$ is more activated than $L_{i}^{non-plto}$ . The two activation diagrams are up-sampled to the initial input size. The direct intensity differences will be figured out in a regular range $D_{i}=\left|L_{i}^{plto}-L_{i}^{non-plto}\right|$ . Patients who match pixels maps are selected. When X-Grad-CAM generated for all samples is used, a group of matrices $D=\left(D_{1}D_{2}\text{···}D_{\text{n}}\right)$ is generated. Also, here n represents the quantity of samples. D which lies on the class of every specimen is separated into two sets, $D^{plto}$ and $D^{non-plto}$ . The difference is in values between $D^{plto}$ and $D^{non-plto}$ .The difference is assessed by applying the Wilcoxon rank-sum test. Also, this process will yield *p*-values. After statistical tests for all pixels, *p*-values are corrected using the Bonferroni correction. The fields of four methods which make use of adjusted *p*-values < 0.001 are observed which comprise 20 pixels. The process is repeated for all 15 pathways, and the details are shown in Table2.

**TABLE 2:** Key pathways associated with patients with the primary lung tumor symptoms.

Row	Pathway	Gene expression	
		PC1	PC2
4	KAECH_NAIVE_*VS.*_DAY15_EFF_CD8_TCELL_DN	0.0001	0.0008
17	GOLDRATH_EFF_*VS.*_MEMORY_CD8_TCELL_UP	0.0003	0.0001
24	GSE10239_NAIVE_*VS.*_KLRG1INT_EFF_CD8_TCELL_DN	0.0003	0.0002
28	GSE10239_MEMORY_*VS.*_KLRG1INT_EFF_CD8_TCELL_DN	0.0002	0.0008
36	GSE10239_MEMORY_*VS.*_DAY4.5_EFF_CD8_TCELL_DN	0.0002	0.0005
46	GSE10325_LUPUS_CD4_TCELL_*VS.*_LUPUS_MYELOID_DN		0.0002
78	GSE11864_UNTREATED_*VS.*_CSF1_IFNG_IN_MAC_DN		0.0001
143	GSE13229_MATURE_*VS.*_INTMATURE_NKCELL_UP		0.0003
147	GSE13306_TREG_*VS.*_TCONV_LAMINA_PROPRIA_UP		0.0002
159	GSE13306_RA_*VS.*_UNTREATED_MEM_CD4_TCELL_UP		0.0005
180	GSE13484_UNSTIM_*VS.*_12H_YF17D_VACCINE_STIM_PBMC_DN		0.0002
186	GSE13484_12H_*VS.*_3H_YF17D_VACCINE_STIM_PBMC_DN		0.0008
250	GSE14308_TH2_*VS.*_INDUCED_TREG_DN	0.0001	
1359	GSE32423_MEMORY_*VS.*_NAIVE_CD8_TCELL_IL7_UP	0.0002	
1425	GSE360_CTRL_*VS.*_B_MALAYI_HIGH_DOSE_MAC_UP	0.0001	

All 20 pixels in the four hot spots are observed in PCs of RNA demonstration. Several pathways which are loaded in both PC 1 and PC 2 are found. Kaplan–Meier analysis is used to analyze the existing time of two groups which are classified by a median separation of PC values which are in the key pixels.

## Discussion

Lung cancer has become a primary cause of cancer deaths, which annually leads to over one million deaths worldwide. There are over 1.2 million new cases which are diagnosed every year. Lung adenocarcinoma reaches an average five-year survival rate of 15%. Lung adenocarcinoma is the most common type of lung cancer. The primary reason is late examination and the scarcity of late treatments. In this situation, smoking undoubtedly becomes the primary reason why many people have been suffering from lung cancer. About 10% of cases occur in patients who have never smoked. LADCs are becoming a major public health concern. The identification of biomarkers can understand the implicit aggressiveness. It plays a key role in the biology of LADCs. Many articles have found assumed survival-associated biomarkers in LADCs. They make it by using the omics platform. The analysis of data which come from individual articles nowadays lacks the capability to find strong biomarkers that are able to make medical decisions in LADCs. United analysis which are about omics data is able to offer insightful information. This information is about the complicated biology and molecular nonuniformity of cancer. Lately, ATT-CNNs have got a huge achievement. Second, applying ATT-CNNs in bioinformatics problems and biological data shown in non-grid structures is a limitation. The application of deep learning techniques of biological data is another limitation. It is put into the interpretability which is in the trained model. Also, it is seen as a “black box.” We recently have generated a conception of “pathway image.” Our goal was to show omics data which made us to adapt ATT-CNNs in biological pathway analysis. Pathways, which are in a connected activity, are put more strongly in the pixel. It can fully leverage ATT-CNN capabilities skillfully which is connected to pixel activity. Also, the ATT-CNN model which is on the united omics data is at the pixel level that remarkably improves predictive performance. Also, it adds co-complemented message to the model. Possibility of the pathway-level analysis that removes noisy information is high. It may lie in individual omics type. This represents every pathway which is seen as a linear collection of related genes. The adoption of X-Grad-CAM and the ATT-CNN model skillfully make biological interpretations, which identify pathways linked with survival in LADC patients. The simplicity and efficiency of the ATT-CNN modeling and X-Grad-CAM lie in biological explanation (Zheng et al., 2017). In this article, the pathway image is for every specimen. Also, its size is linked with 2,200 pathways which are in rows and a number of PCs that are predefined in columns (Zhou et al., 2020). There are missing genes in the omics data because of the dimensional reduction of each pathway. PCA, which releases the difficulty, figures out the missing data shown without those genes. The resulting pathway which is based on the ATT-CNN model effectively predicts PLTO for LADC patients. When omics types are used, it reaches an average AUC of 0.783. This process is applied at the first two PCs for omics type. When omics type is applied, similar performance is performed with an average AUC of 0.769. In conclusion, the application of omics data has a better performance. The biological explanation of the class activation maps is produced. It shows the intensity difference in the two activation diagrams for every sample. Otherwise, the map which applies an X-Grad-CAM technique is derived. There are differences between PLTO and non-PLTO in statistical analysis. Its goal was to find a special pixel on the difference diagram. Although Path-ATT-CNN has demonstrated effective performances in predicting performance, there are some biological problems in processing biological data by the ATT-CNN model. The speed of processing biological issues would be smaller because of too many network layers. Applying the back-propagation method will make adjusting parameters of the input layer slowly. First, applying SGD methods will make trained data restrain local minimum and not global minimum. Second, pooling layers may lose much valuable information and ignore connection of locality and entirety. Third, because of feature extraction, it put a black box in an improvement of network function. This can be further developed in the future. It will be a future extension which includes more closer configuration. It is an essential impact to predict illness.

## Conclusions

In this article, Path-ATT-CNN is designed. The method starts from the concept of “pathway image,” that generates application of omics data. Through this model, we can remarkably learn about metabolic pathways. The good pathway is helpful to patients who can learn about their body mechanism. It is able to predict patient’s lung cancer situation better than any other methods. Then, the use of X-Grad-CAM which is directly applied on the pathway image makes the identification of a plausible pathway. In conclusion, this article shows the future potential to use ATT-CNNs on omics data and X-Grad-CAM. In this way, we can detect more complicated biological correlates of disease disadvantages. We also found a key pathway. There is a limitation to the model, that is, the identification of identical pathways that need closed alignment of the pathways. This can be improved in the next 10 years.

## Data Availability

The original contributions presented in the study are included in the article/supplementary material; further inquiries can be directed to the corresponding author.

## References

[B1] Ampie L. Woolf E. C. Dardis C. (2015). Immunotherapeutic Advancements for Glioblastoma. Front. Oncol. 5, 12. 10.3389/fonc.2015.00012 25688335 PMC4310287

[B2] Bengio Y. Goodfellow I. Courville A. (2017). Deep Learning. MA, USA: MIT press Cambridge.

[B3] Cai W. Wei Z. (2020). Remote Sensing Image Classification Based on a Cross-Attention Mechanism and Graph Convolution. IEEE Geoscience Remote Sens. Lett. 19. 10.1109/LGRS.2020.3026587

[B4] Chen L. Chen J. Hajimirsadeghi H. Mori G. (2020). Adapting Grad-CAM for Embedding Networks. arxiv, 2783–2792.

[B5] Du J. Gui L. He Y. Xu R. Wang X. (2019). Convolution-Based Neural Attention with Applications to Sentiment Classification. IEEE Access 7, 27983–27992. 10.1109/access.2019.2900335

[B6] Evans R. Jumper J. Kirkpatrick J. Sifre L. Green T. Qinv C. (2018). De Novo structure Prediction with Deeplearning Based Scoring. Annu. Rev. Biochem. 77, 363–382.

[B7] Goldstein A. Veres P. Burns E. Briggs M. S. Hamburg R. Kocevski D. (2017). An Ordinary Short Gamma-Ray Burst with Extraordinary Implications: Fermi -GBM Detection of GRB 170817A. Astrophysical J. Lett. 848 (2), L14. 10.3847/2041-8213/aa8f41

[B8] Golestaneh S. A. Karam L. J. “Spatially-Varying Blur Detection Based on Multiscale Fused and Sorted Transform Coefficients of Gradient Magnitudes,” in 2017 IEEE Conference on Computer Vision and Pattern Recognition (CVPR), Honolulu, HI, USA, July 2017, 596–605.

[B9] Learning D. (2020). Deep Learning,” *High-Dimensional Fuzzy Clustering* . Chicago: Chicago International Breast Course.

[B10] LeCun Y. Bengio Y. Hinton G. (2015). Deep Learning. Nature 521 (7553), 436–444. 10.1038/nature14539 26017442

[B11] Li Z. Liu F. Yang W. Peng S. Zhou J. “A Survey of Convolutional Neural Networks: Analysis, Applications, and Prospects,” in Proceedings of the IEEE Transactions on Neural Networks and Learning Systems, June 2021. 10.1109/tnnls.2021.3084827 34111009

[B12] Liu J. Zha Z.-J. Hong R. Wang M. Zhang Y. “Deep Adversarial Graph Attention Convolution Network for Text-Based Person Search,” in Proceedings of the 27th ACM International Conference on Multimedia, October 2019, 665–673.

[B13] Lomonaco V. Pellegrini L. Rodriguez P. Caccia M. She Q. Chen Y. (2022). CVPR 2020 Continual Learning in Computer Vision Competition: Approaches, Results, Current Challenges and Future Directions. Artif. Intell. 303, 103635. 10.1016/j.artint.2021.103635

[B14] Nandhini Abirami R. Durai Raj Vincent P. Srinivasan K. Tariq U. Chang C.-Y. (2021). Deep CNN and Deep GAN in Computational Visual Perception-Driven Image Analysis. Complexity 2021. 10.1155/2021/5541134

[B15] Wang L. Huang Y. Hou Y. Zhang S. Shan J. “Graph Attention Convolution for Point Cloud Semantic Segmentation,” in Proceedings of the 2019 IEEE/CVF Conference on Computer Vision and Pattern Recognition (CVPR), Long Beach, CA, USA, June 2019, 10296–10305.

[B16] Wirsching H.-G. Weller M. (2017). Glioblastoma,” *Malignant Brain Tumors* . Hong Kong: Glioblastoma, 265–288. 10.1007/978-3-319-49864-5_18

[B17] Wu Z. Pan S. Chen F. Long G. Zhang C. Yu P. S. (2020). A Comprehensive Survey on Graph Neural Networks. IEEE Trans. Neural Netw. Learn Syst. 32 (1), 4–24. 10.1109/TNNLS.2020.2978386 32217482

[B18] Wu J. (2017). Introduction to Convolutional Neural Networks. Natl. Key Lab Nov. Softw. Technol. 5, 495.

[B19] Yuan L. Huang D.-S. (2019). A Network-Guided Association Mapping Approach from DNA Methylation to Disease. Sci. Rep. 9 (1), 1–16. 10.1038/s41598-019-42010-6 30944378 PMC6447594

[B20] Yuan L. Zheng C.-H. Xia J.-F. Huang D.-S. (2015). Module Based Differential Coexpression Analysis Method for Type 2 Diabetes. BioMed Res. Int. 2015, 836929. 10.1155/2015/836929 26339648 PMC4538423

[B21] Yuan L. Zhu L. Guo W. L. Zhou X. Zhang Y. Huang Z. (2016). Nonconvex Penalty Based Low-Rank Representation and Sparse Regression for eQTL Mapping. IEEE/ACM Trans. Comput. Biol. Bioinform 14 (5), 1154–1164. 10.1109/TCBB.2016.2609420 28114074

[B22] Yuan L. Yuan C.-A. Huang D.-S. (2017). FAACOSE: A Fast Adaptive Ant Colony Optimization Algorithm for Detecting SNP Epistasis. Complexity 2017. 10.1155/2017/5024867

[B23] Yuan L. Guo L. H. Yuan C. A. Zhang Y. H. Han K. Nandi A. (2018). Integration of Multi-Omics Data for Gene Regulatory Network Inference and Application to Breast Cancer. IEEE/ACM Trans. Comput. Biol. Bioinform 16 (3), 782–791. 10.1109/TCBB.2018.2866836 30137012

[B24] Yuan L. Sun T. Zhao J. Shen Z. (2021). A Novel Computational Framework to Predict Disease-Related Copy Number Variations by Integrating Multiple Data Sources. Front. Genet. 12, 696956. 10.3389/fgene.2021.696956 34267783 PMC8276077

[B25] Yuan L. Zhao J. Sun T. Shen Z. (2021). A Machine Learning Framework that Integrates Multi-Omics Data Predicts Cancer-Related LncRNAs. BMC Bioinforma. 22 (1), 1–18. 10.1186/s12859-021-04256-8 PMC8210375 34134612

[B26] Yun S. Jeong M. Kim R. Kang J. Kim H. J. (2019). Graph Transformer Networks. Adv. neural Inf. Process. Syst. 32.

[B27] Zheng H. Fu J. Mei T. Luo J. “Learning Multi-Attention Convolutional Neural Network for Fine-Grained Image Recognition,” in Proceedings of the 2017 IEEE International Conference on Computer Vision (ICCV), Venice, Italy, October 2017, 5209–5217.

[B28] Zhou J. Cui G. Hu S. Zhang Z. Yang C. Liu Z. (2020). Graph Neural Networks: A Review of Methods and Applications. AI Open 1, 57–81. 10.1016/j.aiopen.2021.01.001

